# A Two-Day Acceptance and Commitment Therapy (ACT) Workshop Increases Presence and Work Functioning in Healthcare Workers

**DOI:** 10.3389/fpsyt.2020.00861

**Published:** 2020-10-05

**Authors:** Rainer Gaupp, Marc Walter, Klaus Bader, Charles Benoy, Undine E. Lang

**Affiliations:** ^1^ University Psychiatric Clinics (UPK) Basel, Basel, Switzerland; ^2^ University of Basel, Basel, Switzerland

**Keywords:** stress, work, costs, ACT, acceptance commitment therapy, depression, employees, mental health

## Abstract

**Background:**

In this controlled naturalistic study performed in healthcare workers we examined the effect of a two-day acceptance commitment therapy (ACT) workshop on work presence and productivity, i.e. the influence the workshop had on treatment efficacy in a routine hospital care setting.

**Aim:**

To examine the influence of ACT on productivity and presence in healthcare workers.

**Method:**

Study participants were all healthcare workers (nurses, medical doctors, physiotherapists, social workers, and art therapists) of four inpatient wards for depression. Half of the healthcare workers attended the workshop. Measures were evaluated 3 months after the intervention in the study participants and the patients treated by them in the same time period.

**Results:**

A significantly higher treatment efficacy [as measured with HoNOS (Health of the nation outcome scales) change in the patients treated by the participants] has been observed in the healthcare workers who attended the ACT workshop when compared to the control group who did not attend the workshop. Moreover, the work presence of the participants of the ACT workshop was increased when compared with the time period before the intervention and with the presence of the control group. A cost analysis showed that ACT workshops lead to a significant return of investment for the employer as the costs for the workshop were ten times compensated by the increase of work presence in participants of the workshop.

**Conclusion:**

These findings provide support that ACT interventions motivate healthcare workers to work and increase their patients’ treatment quality. To our knowledge this is the first study showing an ACT workshop in healthcare workers can influence HoNOS outcome in the treated patients.

## Introduction

In Europe, the costs of 12 most relevant groups of mental health disorders were conservatively estimated on 386 billion Euros per year (1). About half of these costs were caused by anxiety disorders, depressive disorders, and addiction and more than half of these costs are paid by the employer due to productivity losses (1). In Switzerland, the costs of depression at the workplace have been estimated on 8 billion Euros per year (2). The ultimate payer for the costs is the employer and not the health system since the majority of the costs are not incurred within the health care systems but in the workplace. Indeed, in Switzerland a study estimated, that the mean total direct costs for the treatment of depression range from 3.561 to 16.240 Euros from mild to severe depression per person per year (2). The mean indirect costs that are due to workdays lost range from 8.730 to 16.669 Euros for mild to severe depression per person per year (2). Therefore, at least in Switzerland, the treatment of depression is connected to lower costs than the amount of money the employer pays for the disorder. In conclusion, the economic burden of mental health disorders is high, i.e. the relative returns from investing in mental health to fight depression and anxiety lead to a four-fold return in better health and ability to work of every dollar invested (3, 4). Especially healthcare workers experience high rates of burn out, anxiety disorders, stress, and depression, e.g. half of the physicians at least in America experience symptoms of burn out and this situation even seems to worsen over the last years (5). Workplace conditions that compromise mental health in the healthcare system are especially excessive workloads, increase of bureaucracy, working in emotionally-charged situations, stigma against seeking care, low job control and high job demand, an imbalance between effort and reward and night shifts (4). In conclusion, healthcare staff has been consistently shown to experience above average rates of mental health problems. Mental health disorders in psychiatric staff again affect the quality of patient care, patient satisfaction, and organizational success. Indeed, the mental health of healthcare workers has been reported to be associated with patient safety outcomes, therapeutic incidents, quality of care, patient satisfaction, medical errors, and infections (4, 6–9). In contrast to a huge financial and qualitative damage caused by these disorders, there is only a small proportion of clinically distressed workers that gain access to evidence-based psychotherapeutic interventions (4).

Cognitive behavioral therapy programs that focus on mental health conditions in work-related environment have a strong level of evidence. They reduce presenteeism (presenteeism relates to working employees with reduced productivity due to illness), absenteeism, work disability costs, and loss of time (10). Moreover, work functioning has been shown to be improved by work-related cognitive behavioral programs (11). In contrast, it has been shown recently that non-specific workplace wellness programs did not succeed in terms of clinical measures of health and employment outcomes after 18 months (12). This was shown in 28.937 employees in a recent study including 20 primary control worksites of a large US warehouse retail company (11).

Acceptance and Commitment Therapy (ACT) might be successful as transdiagnostic group-based program because individuals on sick leave often have combinations of health complaints. Indeed, they suffer commonly from multiple symptom disorders and medically unexplained physical symptoms (12, 13). In 106 Swedish social workers, a brief stress management intervention based on the principles of ACT was performed. In this study, the perceived clinical stress was reduced in participants with high stress levels (14). Furthermore in 311 local government employees, where a stress management training and mindfulness and value-based skills have been implemented, a significant clinical improvement has been shown in initially distressed participants of the study (15).

ACT reduces distress associated with a range of physical and mental health difficulties (16). It uses a transdiagnostic framework and is successful in the treatment of anxiety and depression (17), pain (18), addiction (19), obsessive compulsive disorder (20), irritable bowel syndrome (21), diabetes, epilepsy (22), and cancer (23). ACT is based on a dimensional understanding of psychiatric disorders and therefore does not necessarily require categorical diagnoses and diagnostic screening. In this context ACT meets the demand for a treatment of comorbidity in affected populations which is essential as studied disabled populations at the work place display a high symptom overlap. Indeed, ACT might be particularly sufficient as it was conceptualized especially for comorbidity, treatment resistant situations, and disorders with complex symptoms (24). While there is substantial research literature available on ACT in psychiatric and somatic patients, there are a small number of workplace-based intervention studies involving healthy workers (25). Efforts to improve employee health and productivity have been hampered by the failure of early detection of psychiatric diagnoses, the stigma of psychiatry and psychiatric treatment and compartmentalization of medical costs. In this context, an ACT intervention in healthy and not yet diagnosed or screened or selected populations might be successful and effective.

Whilst there are clear insights into the effectiveness of ACT in different treatment settings and diagnostic groups in patients, we are not aware of a study where in a naturalistic design, i.e. without diagnostic evaluation and classification of the study participants, the cost-effectiveness of an ACT intervention in the workplace has been evaluated. Moreover, the influence of ACT on treatment outcomes, i.e. the HoNOS scale (Health of the Nation Outcome Scales) has not been evaluated yet. Accordingly, in our study we were interested in the influence of ACT on 1.) lost time, i.e. the amount of time spent away from the workplace (sick leave over the given time period); 2.) work functioning, i.e. the ACT-related increase of productivity as measured with a given patient outcome parameter (HoNOS) and 3.) costs of the intervention, i.e. costs of the absence rates versus costs of the workshop and its compensation time.

## Materials and Methods

### Study Design

A controlled naturalistic study was performed. For this study we compared findings of four wards of the same hospital, all with a focus on depression. Two wards, i.e. the intervention group (IG) received a 2-day workshop on ACT between May and October 2018. The control group (CG) did not receive any specific training during the study period. In both groups we measured sick leave hours and therapeutic outcome (HoNOS) at baseline (T0) and 3 months after the intervention (T1). There were no inclusion or exclusion criteria for attending the ACT intervention or participating in the research project, as the workshop was part of a routine and integral part of an organization’s qualification program which was obligatory for all members of the staff of the two treatment teams.

### Participants

All participants were employees of the University Psychiatric Clinics (UPK) in Basel. During the period of the study, a total of 28 employees attended an ACT workshop (IG). The majority (79%) of the IG was female. Twenty-two (79%) were registered nurses, 6 (21%) were nursing assistants. The mean age of the participants was 43.8 years (SD = 9.61). Two other depression wards were used as the control group (n = 30). The CG was comparable to the IG with regard to age and sex. Mean age was 45.0 years (SD = 12.12), 73% were female. The qualification of the participants in the CG was slightly different when compared to the IG, as 93% were registered nurses and only 7% were nursing assistants. **Table 1** shows the sociodemographic data of the sample.

**Table 1:** Sociodemographic data of the sample.

		Intervention group (IG)n = 28		Control group (CG)n = 30	
Age (years)		43.8 (SD = 9.61)		45.9 (SD = 12.12)	
Gender	female	78.6%	n = 22	73.3%	n = 22
	male	21.4%	n = 6	26.7%	n = 8
Professional qualification	Registered nurse	78.6%	n = 22	93.3%	n = 28
	Nursing assistant	21.4%	n = 6	6.7%	n = 2

### Patients

Patients routine data were evaluated in the same time period. Eighty-one patients were included in the intervention group and 91 patients in the control group. Patients in the IG were significantly [t(171) = 7.042, p < 0.001, d = 1.20] older (mean = 58.6, SD = 15.5) when compared to patients in the CG (mean = 42.0, SD = 11.8). Gender distribution was comparable (IG: 58.5% female; CG: 53.8% female), as was the most common ICD diagnosis (F3, affective disorders in 72.8% of the IG and 79.1% of the CG). **Table 2** displays the sociodemographic data and principle diagnosis of the evaluated patient groups.

**Table 2:** Sociodemographic data and principle diagnosis of the evaluated patients.

		Intervention groupn = 81		Control groupn = 91	
Age (years)		58.6 (SD = 15.5)		42.0 (SD = 11.8)	
Gender	female	58.0%	n = 47	53.8%	n = 49
	male	42.0%	n = 34	46.2%	n = 42
Principal diagnosis	F0 (Organic, including symptomatic, mental disorders)	6.2%	n = 5	1.1%	n = 1
	F1 (Mental and behavioral disorders due to psychoactive substance use)	6.2%	n = 5	6.6%	n = 6
	F2 (Schizophrenia, schizotypal, and delusional disorders)	6.2%	n = 5	2.2%	n = 2
	F3 (affective disorders)	72.8%	n = 59	79.1%	n = 72
	F4 (Neurotic, stress-related, and somatoform disorders)	7.4%	n = 6	7.7%	n = 7
	F6 (Disorders of adult personality and behavior)	0.0%	n = 0	3.3%	n = 3
	Missing	1.2%	n = 1	0.0%	n = 0

### Intervention

The two-day ACT workshop was being delivered by the leading psychologists of the department, i.e. experienced ACT clinicians, between 9am–5pm. Groups included between 8 and 16 participants. The workshop evaluated in this study was delivered by an in-house ACT therapist, who had extensive experience in delivering individual and group psychotherapy and supervision. Participants were introduced to various techniques. Firstly, the concept of unhelpful thoughts was explained to them and a risk-benefit analyses of psychological impediments. Moreover, mindfulness based practices have been introduced, cognitive defusion was explained, personal values have been evaluated and strategies developed to engage in valued action. In conclusion, the trainer focused on the six main ACT approaches and metaphors (26). After the workshop had been delivered to the participants, individual nurses have been encouraged to assist in a weekly performed ACT group, which had been implemented for the ACT based treatment of patients on both units. Towards the end of the workshop, participants were invited to reflect and share within the group how they might transfer the learning, and further cultivate mindfulness and valuing skills, in their daily lives. 

### Measures

#### Health of Nation Outcome Scales (HoNOS)

For routine outcome measures assessments of patients with severe mental illness, the HoNOS (27) has been used in Switzerland in all psychiatric hospitals. The HoNOS comprises 12 items, each with five response options (scoring range is 0–48), and has good psychometric properties (28, 29). Outcome on clinical problems and psycho-social functioning is assessed by comparing pre-test and post-test total scores on the HoNOS for each patient. The simplest, most straightforward and most commonly used outcome indicator in treatment outcome research is the mean delta from pretest to posttest score.

#### Sick Leave Hours

We used data of the hospital wide attendance recording system and extracted the registered sick leave hours in all four wards for T0 and T1. One day of sick leave is equal 8 h and 24 min. We summed up the time for the intervention and control group.

### Data Analyses

Data were analyzed with IBM SPSS Statistics 26 (IBM SPSS Statistics for Windows, Version 26.0. Armonk, NY : IBM Corp.). Data were analyzed in two stages. First, we examined the effects of the ACT intervention on employees’ presence and cost factors for the employer across the 3-month evaluation period. Secondly, we evaluated the therapeutic progress and therapy effects using the HoNOS scale as a “productivity measure”. Chi-square tests were used for categorical variables. As basic assumptions for Analysis of covariances (ANCOVA) were met and covariates (except for age) were independent, we conducted ANCOVA to test the effects on HoNOS. In the analysis we adjusted for symptom severity at T0, age and sex of the patient and type of discharge. Prior to data analysis, cases with missing values >30% were excluded (30). For the remaining data, Little’s Missing Completely At Random (MCAR) test was non-significant [χ2 (2) = 4.866, p = .088], indicating that the data was indeed missing at random. Hence, missing HoNOS values were imputed using the expectation maximization algorithm (31).

## Results

### Work Presence and Costs of the Intervention

At T0, the total amount of sick leave hours was comparable in the intervention (478 h) and control group (486 h). In the 3 months after the intervention, the amount of sick leave hours dropped to 391 h in the intervention group, but increased to 643 h in the control group [χ2 (1, n = 58) = 28.12, p <.001]. Based on these figures, the gross costs of the intervention are more than amortized within three months. As continuous education is mandatory at our hospital, the costs of the intervention consist of working time of the workshop faculty only (two trainers with 20 h each).

### Productivity/Treatment Outcome

**Figure 1:**
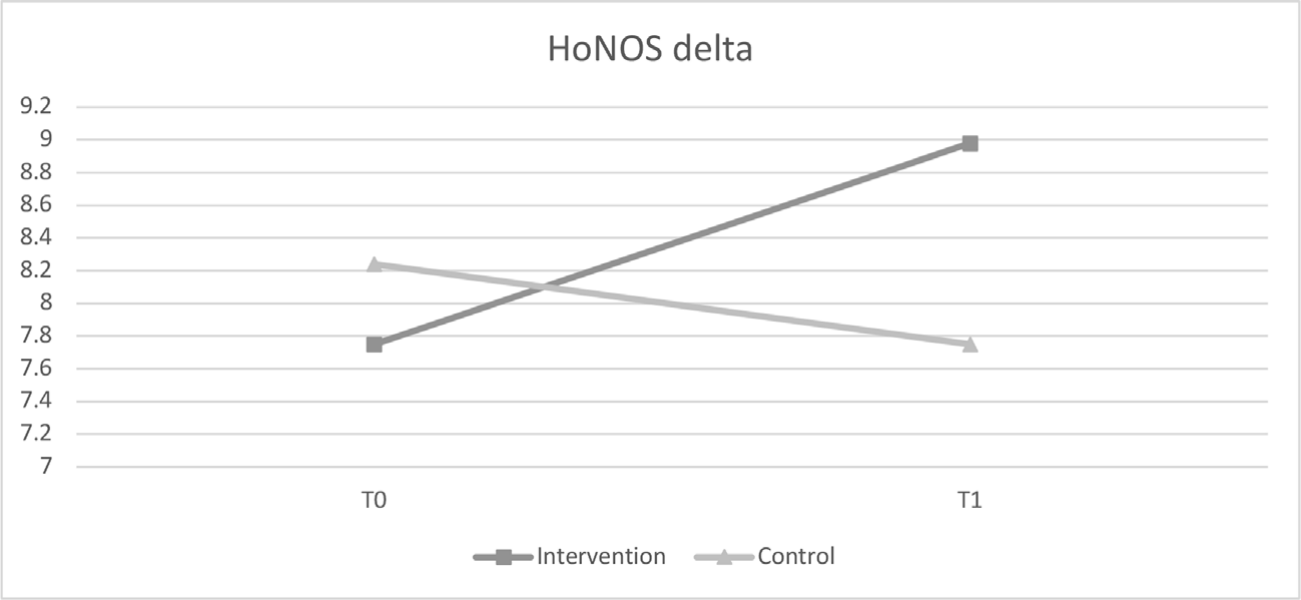
Comparison of HoNOS delta values before and after intervention.

Results of the HoNOS comparisons support our hypothesis that professionals trained in ACT could show improved performance or productivity: A two-way ANCOVA revealed a minor, yet statistically significant, interaction between time and group, controlling for sex, type of discharge, age and symptom severity at time of hospitalization [*F* (1;163) = 4.451, *p* = .036, partial η^2^= .03]. The achieved HoNOS delta in the intervention group improved significantly in the intervention group (T0: 7.72 vs. T1: 8.98), whereas there was a slight reduction of the achieved HoNOS delta in the control group (T0: 8.24 vs. T1: 7.75) (**Table 3**; **Figure 1**).

**Table 3:** Descriptive statistics and p-values for outcome measures.

Measure	T0		T1		p
	IG	CG	IG	CG	
Sick Leave hours	478	486	391	643	<.001
HoNOS delta	7.72 (SD = 4.80)	8.24 (SD = 6.28)	8.98 (SD = 4.61)	7.75 (SD = 6.55)	.036

IG, intervention group; CG, control group.

## Discussion

Our results indicate that ACT was effective in improving the general work presence in a sample of employees across a three months evaluation period and when compared with a control condition. Moreover, the work productivity, as measured with a patient outcome (HoNOS) significantly improved in the three months period and was increased when compared with the control condition. Because of the high efficacy and brevity of the intervention, the costs of the intervention were significantly lower when compared with the losses in the control group; i.e. the intervention was cost effective. In this context ACT might be an appropriate tool to increase treatment quality and efficacy at least in depression and in transdiagnostic wards in psychiatric hospitals.

Our data are in line with a recent study, where ACT reduced levels of work stress in 91 individuals in a randomized controlled clinical trial (32). Moreover, according to our findings a university-based ACT training has been shown to increase self-care, clinical competencies, and therapist skill development in clinical psychology trainees (33). Our data are further in line with a study, where 90 volunteers in a media organization were randomly allocated to an ACT group, an Innovation Promotion Program and a waitlist control group. In this study, both interventions showed improvements in mental health, presence and work-related variables (34). Additionally, another study showed that an ACT intervention decreased experiential avoidance, perceived stress, and burnout in 113 nursing students (15). In line with our data it has also been shown that an ACT intervention led to significantly greater reduction in distress in staff working with individuals with intellectual disability which was maintained within a six-week period (35). In addition, the employability has been increased in patients with mental illness and/or chronic pain by an ACT intervention in a randomized controlled trial (36).

Moreover, our findings indicate that the beneficial effects of an ACT workshop on employees’ in a psychiatric hospital were linked to improvements in specific treatment skills, i.e. help people pursue and expand personally valued patterns of behavior. Indeed, ACT was successful in expanding the therapeutic benefits of the participants as measured objectively in the work performance of the study participants, i.e. the HoNOS change in the treated patients, which is an indicator for therapeutic success at least in Switzerland, where it is measured in all hospitals as a qualitative and financial outcome variable.

In a new report of 2010, European costs of mental health disorders were estimated on 798 billion Euros (37). In this report direct health care cost were 37%, direct non-medical cost 23%, and indirect cost 40%. Total annual costs per disorder were 65.7 billion Euros for addiction, 74.4 billion Euros for anxiety, 113.4 billion Euros for depression, 27.3 Euros for personality disorders, 93.9 Euros for psychotic disorders, 35.4 Euros for sleep disorders, and 21.2 Euros for somatoform disorders. These data reveal that psychiatric disorders overall are much more costly than previously estimated and they constitute a major health economic challenge for Europe (31). In another corresponding study, conditions with the highest estimated daily productivity loss and annual cost per person were chronic back pain, mental illness, general anxiety, migraines or severe headaches, neck pain, and depression (32). In our study, we also question the return of investment concerning the influence of the workshop on presence on the workspace. In this context our data are in line with a recent study, where the cost-effectiveness of ACT for employees on sickness absence has been considered favorable in terms of cost-utility analyses (38).

However, these rising costs of healthcare employees increase the direct cost burden of the medical health system and presentism is a relevant factor in this context. Presenteeism, which is commonly referred to as an employee at work who has impaired productivity due to health considerations, has been identified as an indirect but relevant factor influencing productivity and human capitol (39). Especially in psychiatry the human capitol is necessary to help patients, to be optimistic, to build constructive therapeutic relationships and to prevent patients from fatal complications.

However, in our study we posed the question if ACT is effective in a naturalistic setting, where all healthcare workers of a team are included, without stratifying them at baseline in order to exclude non-distressed individuals. This concept makes sense as in daily business screening might lead to drop out rates as employers might not be willing to be “diagnosed” and “treated” but rather be a part of a qualification program of the whole treatment team. This makes sense as the rate of psychiatric and somatic disorders—which can all be treated by ACT—are high in the general working population. In this context, the inclusion of all potentially suffering individuals might be more effective than excluding the healthy individuals. We promote that an integration of highly effective treatment options irrespective of diagnostic or disease entities in the workplace is an efficacious strategy to save costs, motivate workers, and increase work productivity. In this context the aim might not primarily be to screen diagnoses on the workplace and reference employees to a psychiatric treatment but implement high quality and highly evidenced transdiagnostic programs payed by the employer. A transdiagnostic approach to work disability may be desirable also from a participant perspective of view since loss of work capacity is equally validated regard-less of cause, rather than dichotomized into somatic or mental type and help to avoid treatment barriers associated with stigma of psychiatric disorders and psychiatry as a discipline.

### Limitations

It is important to note also limitations in the design of the current study. Our sample size is relatively small, and although we were able to make use of a control group, who has not been qualified in ACT, participants were not randomly allocated to the condition. Moreover, an effect of seasonal variation might not be ruled out and we did not evaluate protocol adherence of the professionals in the intervention group. However, we followed a naturalistic design and it might also be an advantage that all members of the treatment teams were included in the ACT and control condition. Generalizability of the results may be limited, as the sample included only healthcare professionals from two professions, all of them working in the same hospital. Another limitation is that we did not focus on the psychopathological state of the health workers and did not measure perceived stress or other health outcome parameters of the employees. In this context the influence of our intervention on known ACT variables as mindfulness and acceptance processes, values and committed action elements has not been evaluated in our study.

## Conclusions

In conclusion, the present study provides preliminary practice-based evidence that a brief ACT intervention can be effective in improving productivity, employers’ costs, and work presence of healthcare employees. We recommend implementing work-focused ACT interventions to help to increase work time and motivation at work and decrease costs associated with absence from work (3). Practitioners should consider implementing these programs to help improve work functioning, work quality, and patient outcome in a clinical setting (3). To integrate disease prevention with disease management in different work environments, we propose a procedure where all members of a treatment team are being skilled and show in this paper that this is still cost effective and might discharge the health care system and prevent disease progress. We propose that ACT as a transdiagnostic program performed in all staff members of a treatment team irrespective of their individual health status might be a successful and efficient procedure to reach the distressed individual, to increase work productivity and work presence, and to decrease employers costs. Moreover, we show for the first time that treatment effects on HoNOS outcome in the treated patients.

## Data Availability Statement

The raw data supporting the conclusions of this article will be made available by the authors, without undue reservation, to any qualified researcher.

## Ethics Statement

Ethical review and approval was not required for the study on human participants in accordance with the local legislation and institutional requirements. Written informed consent from the patients/ participants was not required to participate in this study in accordance with the national legislation and the institutional requirements.

## Author Contributions

KB and CB invented the whole ACT concept and established it in Basel and trained the trainer and therapists in this approach. MW is Co-Chief und UL is Chief of the department where the organizational procedures were implemented. UL wrote the manuscript together with RG and MW. RG and MW made the statistic measurements. All authors contributed to the article and approved the submitted version.

## Conflict of Interest

The authors declare that the research was conducted in the absence of any commercial or financial relationships that could be construed as a potential conflict of interest.
